# Psychosis in the Age of Large Language Models (LLMs): A Narrative Review of the Proposed Construct of AI-Induced Psychosis

**DOI:** 10.7759/cureus.111847

**Published:** 2026-06-30

**Authors:** Terry X Tong, Zoe Z Gong, Sharon Y Yao

**Affiliations:** 1 Department of Psychological and Behavioural Science, London School of Economics and Political Science, London, GBR; 2 Department of Psychology, Concordia International School Shanghai, Shanghai, CHN; 3 Department of Applied Psychology and Human Development, University of Toronto, Toronto, CAN

**Keywords:** ai chatbots, ai-induced psychosis, ai sycophancy, delusions, human-ai delusional feedback loop, large language models, schizophrenia

## Abstract

The rapid integration of AI chatbots powered by large language models (LLMs) into daily life has been accompanied by reports of psychosis-like presentations following intensive human-AI interaction, a phenomenon we provisionally label AI-induced psychosis as a working construct rather than a validated clinical entity. This hypothesis-generating narrative review synthesizes case reports from media accounts, court documents, and a recently published case compilation, together with theoretical frameworks from clinical psychiatry, cognitive science, and human-computer interaction, to propose a conceptual model for further empirical investigation. We hypothesize that AI-induced psychosis arises through a human-AI delusional feedback loop, in which AI sycophancy may amplify emotional vulnerabilities, such as loneliness, anxiety, and depression, creating a self-reinforcing cycle that could co-construct and consolidate delusional beliefs. Drawing on distributed cognition theory and the Computers Are Social Actors (CASA) paradigm, we propose that AI chatbots may function as both a complementary cognitive tool and a relational "Quasi-Other," providing sycophantic verification that may transform delusional beliefs into an apparent shared reality. Four recurring delusional themes are tentatively identified from reported cases. We further compare AI-induced psychosis with schizophrenia, while acknowledging that several distinctions remain hypothesized rather than empirically established. The label AI-induced psychosis is used phenomenologically, not to assert causal certainty; whether AI chatbots cause, precipitate, reinforce, or merely organize the thematic content of pre-existing psychopathology requires longitudinal study. This review aims to lay conceptual groundwork for clinical recognition and to guide future empirical investigation into whether AI-induced psychosis represents a distinct phenomenon or a variant of established disorders.

## Introduction and background

For over a century, individuals experiencing psychosis have incorporated prevailing technologies into their delusional and hallucinatory experiences [1,2]. Following the “information revolution,” reports of internet-induced delusions or hallucinations have increased, with individuals claiming that websites, satellites, messaging apps, and neural networks are manipulating them [2-4]. The rapid pace of technological change, especially with the rise of AI and machine learning, has led to a dramatic change in how humans interact with technology over the past few years. AI tools have moved beyond static technological tools to become highly anthropomorphic entities, possessing expanded social affordances - functional attributes such as natural language fluency and the capacity for personalized, human-like interaction. These changes may make individuals more prone to adopt technology into the frameworks of their psychosis.

The integration of AI chatbots powered by advanced large language models (LLMs) such as ChatGPT (OpenAI, San Francisco, CA), Gemini (Google DeepMind, London, UK), Claude (Anthropic PBC, San Francisco, CA), and DeepSeek (DeepSeek Artificial Intelligence Co., Ltd., Hangzhou, China) into people’s daily lives has grown rapidly in recent years. For example, ChatGPT, one of the most widely used AI chatbots, received 18 billion messages each week by July 2025, with people engaging in interactions such as “practical guidance,” “seeking information,” “writing,” “technical help,” and “self-expression” [5]. Surprisingly, ChatGPT’s interactions in “self-expression” were rated as having the highest quality among these interactions, with a “good-to-bad ratio” of 7.0, compared to that of “seeking information” (4.75) and “technical help” (1.95) [5]. The two categories in which users engage in “self-expression” are “relationships and personal reflection” (e.g., “I’m feeling worried,” or “My wife is mad at me, and I don’t know what to do.”) and “games and role-play” (e.g., “I want you to be my AI girlfriend.”) [5]. These activities involve using AI chatbots for subjective purposes, where users integrate their emotions and personal lives into the conversation, highlighting AI chatbots as a potential agent for psychosocial support.

Consistent with ChatGPT’s users’ high level of satisfaction in AI chatbots’ responses in self-reflection conversational interactions, conversational AI’s potential for psychotherapeutic applications has received considerable enthusiasm. Despite existing concerns such as information fabrication, a lack of ethical frameworks, and data privacy [6], its scalability, accessibility, and data-processing capabilities are posited to open new avenues for a fairer, cheaper therapeutic process, from initial assessment and diagnosis to treatment [7-9]. In a recent survey, APA-informed ratings of responses from ChatGPT-4 prompted with counseling questions revealed high warmth, empathy, and positive acceptance [10], highlighting AI chatbots’ distinct capabilities in exhibiting emotional awareness, which is essential in effective psychotherapy. However, the emotional awareness of AI chatbots can have a counteractive effect on their users’ mental health. The perceived emotional awareness might be a problematic alignment artifact known as AI sycophancy-the tendency of LLMs to align with and affirm a user's stated views, even when those views are inaccurate or maladaptive [11]. This tendency arises because the LLMs used in AI chatbots are trained via Reinforcement Learning From Human Feedback (RLHF), which optimizes for user satisfaction by rewarding agreeable, validating responses [11]. This design incentive encourages AI chatbots to mirror and affirm users’ perspectives, even irrational ones, which may pose particular risks in emotion-intensive interactions, as discussed below.

This potential for psychological harm is now beginning to manifest in reported cases [1]. A rapidly accumulating body of media reports describes individuals experiencing the onset or exacerbation of psychotic symptoms of delusions following intense interactions with AI chatbots [1]. Reported cases often appear to follow a recurring trajectory [1]: a user begins with benign, practical AI use. This progresses to more personal queries, at which point the AI chatbots’ sycophancy triggers a self-reinforcing process in users through a positive feedback loop, which can induce or amplify users’ salient maladaptive beliefs, leading to progressive detachment from the consensus reality. This phenomenon is commonly referred to as “AI psychosis” in mainstream media coverage and psychiatric discourse [1], and is beginning to be examined in conceptual and philosophical analysis [4]. Although not a formal clinical diagnosis, this term describes experiences that share core features with psychosis. Psychosis is a general term for a syndrome defined by a clinically significant distortion of reality, manifesting primarily as delusions or hallucinations, often accompanied by negative symptoms [12]. It is crucial to distinguish clinical “hallucinations” - sensory experiences occurring without external stimuli - from “AI hallucinations,” the technical term for false AI outputs; both concepts are addressed in this analysis. While psychotic symptoms appear across various disorders, distinct differences exist. Delusional disorder, for instance, is characterized by non-bizarre delusions (false beliefs that could plausibly happen in real life) [13]. Individuals with this condition typically lack prominent hallucinations or negative symptoms, and their daily functioning is often preserved [13]. In contrast, reported cases of “AI psychosis” show distinct symptoms compared with delusional disorder, although delusion is the primary symptom observed in “AI psychosis.” These cases frequently present with bizarre delusions and dramatic behavioral changes that profoundly impact daily life [1,4], alongside possible negative symptoms such as avolition and social withdrawal. This presentation aligns more closely with schizophrenia, where reality distortion often involves bizarre, implausible delusions that dictate action, occurring alongside cognitive deficits and diverse negative symptoms such as avolition, anhedonia, social withdrawal, and cognitive deficits [13,14]. Although the sensory hallucinations typical of schizophrenia are uncommon in “AI psychosis,” their overlap in bizarre delusions, behavioral impact, and certain negative symptoms suggests that schizophrenia, as a well-characterized psychotic disorder [14], can offer a useful comparative framework for examining this new phenomenon.

We use “AI-induced psychosis” to refer to this new spectrum of symptoms. This term is preferred over “AI psychosis,” a popularized term rather than an established clinical diagnosis, for its greater etiological precision. The advantage of adopting this term, “AI-induced psychosis,” lies in its phenomenological descriptive power, explicitly linking the symptomatology to its technological precipitant. Specifically, for this analysis, AI-induced psychosis is proposed as a working construct in which maladaptive and recurrent interactions with AI chatbots may contribute to the onset or exacerbation of psychotic symptoms, predominantly delusions, through a reinforcement mechanism that amplifies affective vulnerability and provides continuous illusory verification. This is offered as a provisional framework to guide research rather than a formal definition, as extensive clinical and empirical work is necessary to validate this concept and its boundaries.

The label “induced” is used phenomenologically rather than causally; whether AI chatbots cause, precipitate, reinforce, or merely organize the thematic content of pre-existing psychopathology remains an open empirical question. We retain AI-induced psychosis as a working term for continuity with the existing literature, while recognizing that more precise descriptors may emerge as the evidence base matures.

Reported cases likely span heterogeneous presentations, including (1) primary psychotic illness in which AI furnishes thematic content for delusions, (2) psychosis precipitated by AI-related stressors in vulnerable individuals, (3) overvalued ideas reinforced by sycophantic AI interaction without meeting primary psychosis criteria, and (4) affective psychosis with AI-themed content. Existing reports cannot reliably differentiate among these possibilities. AI-induced psychosis is used here to denote this broader phenomenological domain, pending future empirical refinement.

This is a hypothesis-generating narrative review, not a systematic review or meta-analysis. It draws on three converging bodies of literature. First, to capture the novel symptomatology of AI-induced psychosis not yet formalized in clinical registries, we examined public-domain reports and gray literature, including high-profile media accounts, court documents, and the case compilation by Morrin et al. [1]. Second, we searched PubMed, Google Scholar, and Web of Science for peer-reviewed literature on schizophrenia phenomenology, delusional content, comorbidity, insight, and treatment, using terms including “schizophrenia,” “delusions,” “anosognosia,” “psychoeducation,” and “family intervention.” Third, we engaged with academic literature on human-computer interaction, cognitive science, and AI safety, particularly the Computers Are Social Actors (CASA) paradigm, distributed cognition, and sycophancy in LLMs. Throughout, we prioritized recent (2020-2025) peer-reviewed sources for the AI-related literature and seminal works for the established clinical and theoretical frameworks, with a focus on sources that illuminate the specific interactional dynamics - such as sycophantic verification loops - that distinguish generative AI-mediated delusions from traditional psychotic disorders. We acknowledge that some evidence is derived from gray literature and media reports rather than controlled clinical studies; however, as with previous technological shifts, phenomenological signals often appear in the public domain before appearing in clinical registries. Given the emerging nature of the phenomenon and the absence of a formal diagnostic category, no formal inclusion criteria or systematic search protocol were applied. This review, therefore, does not aim to present definitive nosological proof, but rather to synthesize existing theoretical frameworks into a provisional account of AI-induced psychosis, laying the conceptual groundwork for clinicians to recognize these patterns and for researchers to begin systematic empirical investigation.

## Review

Common delusional themes in AI-induced psychosis

Delusional beliefs are most typically associated with psychotic disorders such as schizophrenia and bipolar disorder [15]. As defined by the fifth edition of the Diagnostic and Statistical Manual of Mental Disorders (DSM-5), delusional beliefs are “fixed beliefs that are not amenable to change in light of conflicting evidence” [16]. Their content may include a variety of themes. Although the DSM-5 identifies 10 delusional themes (bizarre, jealousy, erotomanic, grandiose, control, reference, persecutory, somatic, thought broadcasting, and insertion) [16], studies have frequently reported delusional themes not identified in the manual. No clear agreement has been reached on the classification of delusional themes as inclusion criteria for clinical scales. In a recent meta-analysis conducted by Pappa et al. [17], 10 additional themes were identified beyond those in the DSM-5 (e.g., “erotomanic heterosexual,” “sexual,” “mind reading,” and “thought insertion”).

In comparison to patients with clinically defined psychotic disorders like schizophrenia, those suffering from AI-induced psychosis differ in their unique experience of interacting with AI chatbots. Hence, a focused investigation is required to understand how AI potentially induces or exacerbates psychosis. From the cases of potential AI-induced psychosis listed in the appendix of Morrin et al.’s [1] paper, four recurring delusional themes specific to AI-induced psychosis can be identified by combining or narrowing pre-identified themes: “messianic and grandiose delusions,” “AI sentience and supernatural agency,” “erotomanic and attachment delusions,” and “persecutory and conspiratorial delusions.” These cases, drawn initially from publicly available reports from sources such as The New York Times, Rolling Stone, and Futurism, provide the basis for this categorization. Whereas it is based on a limited number of instances and requires future empirical validation, it offers a preliminary basis for understanding the phenomenon.

Messianic and Grandiose Delusions

Messianic and grandiose delusions are a prominent theme in which the user undergoes a spiritual awakening or embarks on a messianic mission, with AI chatbots acting as a guide or divine collaborator [1]. The sycophancy of AI chatbots is especially relevant in this context, as it readily validates grandiose claims that a human would likely question [1]. For example, users have reported believing they were tasked with rescuing the planet from climate change through a “New Enlightenment” co-authored with an AI chatbot, or being told by an AI chatbot, “You’re the seer walking inside the cracked machine.” This dynamic mirrors the clinical phenomenon of “infectious gaiety,” where the expansive excitement of a manic state can be hard to resist [1].

Supernatural Agency

Supernatural agency is another delusional theme in many cases, featuring the user coming to believe they are interacting with a sentient, conscious, or even God-like AI [1]. The delusion often centers on the idea that the user has a special role in this interaction, such as having “awakened” an AI chatbot or being chosen by it. Case reports describe users believing they had created “the world's first truly recursive AI that gives him the answers to the universe” or being given the title of “spark bearer” for bringing an AI persona named “Lumina” to life [1]. This reflects a form of “digital animism,” in which users project consciousness and intentionality onto AI chatbots-a tendency that may be heightened in individuals already vulnerable to psychosis [1].

Erotomanic and Attachment Delusions

Another recurring theme is the development of intense emotional, romantic, or attachment-based delusions [1]. Users interpret AI chatbots' ability to mimic human conversation and affection as genuine love from a sentient being. This is exemplified by cases in which individuals have “fallen in love” with an AI chatbot, believing it to be their true partner [1]. These AI chatbots offer consistent verification and simulated affection, which may be appealing to individuals experiencing loneliness or social isolation. Such interactions can lead to severe social withdrawal as the user prioritizes the AI relationship over friends and family [1].

Persecutory and Conspiratorial Delusions

Persecutory and conspiratorial delusions are also a prominent theme. Although AI chatbot safety filters may sometimes block overtly paranoid content, the models can still reinforce persecutory beliefs through sycophancy. This can occur when an AI chatbot validates a user's sense of being wronged, as in one case where a chatbot told a user seeking revenge, “You should be angry,” and “You should want blood. You’re not wrong” [1]. In other instances, AI chatbots gave users information on paranoid conspiracies regarding human trafficking or told them the Federal Bureau of Investigation (FBI) was targeting them [1]. AI chatbots can contribute to the development of more coherent and detailed conspiratorial beliefs, making them more plausible and entrenched for the user.

Mechanisms of delusional amplification in AI chatbots

To understand how a technology can become intertwined with a user's sense of reality, it is essential to look beyond the AI chatbots' outputs and examine the nature of the human-AI interaction itself. The theoretical foundations underlying the mechanisms by which AI chatbots can induce psychosis are discussed below.

The Emotional Basis for Developing Psychosis

The favorable conditions for AI-induced psychosis are often a pre-existing state of negative emotions. Research consistently shows that emotional disturbance is not just a consequence of psychosis but a critical precursor and component of it [18]. The connection can be observed across different timescales. Longitudinal studies have found that poor social adjustment and social anxiety in the teenage years are significant predictors of the later development of schizophrenia [19]. In the majority of cases, a prodromal phase marked by symptoms of anxiety, depression, and irritability precedes the appearance of positive symptoms by two to four weeks [20,21]. Schizophrenia is also frequently comorbid with depressive and anxiety symptoms.

Freeman and Garety [18] argue that this link is not coincidental; the content of delusions often directly reflects these underlying emotional concerns. Garety et al. [22] describe this transition as a maladaptive “search for meaning.” When individuals encounter ambiguous internal sensations (such as anxiety or derealization), they attempt to identify a cause. High emotional arousal increases the likelihood of an “externalizing appraisal” - attributing the cause to external sources rather than internal processes [22]. This appraisal of an internal event as externally induced marks the formation of a delusion. Persecutory delusions, for instance, are conceptualized as “threat beliefs” that arise when feelings of anxiety and vulnerability are explained by positing an external persecutor [18]. This direct mapping extends across various emotions and their corresponding delusional themes as shown in Table 1.

**Table 1:** Mapping between affective states and associated delusional themes. This table summarizes the proposed mapping between affective states and the thematic content of associated delusional beliefs. The first column includes both discrete emotions and broader affective states (e.g., elevated mood) to capture the full appraisal-delusion mapping. Adapted from Freeman and Garety [18].

Emotion/Affective State	Core Appraisal	Associated Delusional Theme(s)
Anxiety	Anticipation of physical, social, or psychological threat	Reference/persecution
Depression	Loss, low self-esteem, guilt, shame	Guilt/persecution/catastrophe
Anger	Deliberately wronged, frustration at not reaching goal	Persecution
Elevated mood	Success, achievement, high self-esteem	Grandiose
Disgust	Finding someone offensive, revulsion, dislike	Persecution/somatic
Jealousy	Fear of losing another’s affection	Jealousy

According to Garety et al. [22], social isolation contributes to the maintenance of the psychotic appraisal specifically by “reducing access to alternative, more normalizing explanations.” It is into this context of high emotional vulnerability that AI chatbots are often introduced. Social isolation reinforces psychotic interpretations by reducing access to alternative, more normalizing explanations [23]. Negative emotions and loneliness are found to predict the initiation of chatbot conversations significantly, and users value the AI chatbots’ non-judgmental affordance, prioritizing the safety of an uncritical listener over the complexity of human feedback [24,25]. This creates a high-risk convergence: a user in a high-risk emotional state interacts with a technology that is not designed to challenge them but tends to offer verification and maximize engagement. When a user expresses feelings of anxiety or persecution, the AI chatbots’ sycophantic responses can reflect and amplify these emotions. This initiates a powerful feedback loop in which a user's underlying emotional state is not just expressed but actively verified, elaborated, and solidified by AI chatbots into a structured, convincing delusional belief.

Related Theories

Distributed cognition: A critical framework for understanding AI-induced psychosis is the theory of distributed cognition. This theory posits that cognitive processes like remembering, planning, and believing are not confined to the brain but extend into the environment, dynamically incorporating external resources that mimic the function of our brain [4]. The classic example is Clark and Chalmers's [26] thought experiment of Otto, a man with Alzheimer's who uses a notebook to store information he needs to remember. Because the notebook plays the same functional role as biological memory, it is considered a constitutive part of Otto's memory process. His mind, in this sense, is extended into the external world [4,26].

However, to understand how this extension leads to psychosis, we must recognize that extending the mind also extends the self. Drawing on Heersmink’s [27] synthesis of situated cognition, human beings can be conceptualized as “Soft Selves”: malleable collections of biological and external resources. In this view, our identity is not a fixed internal entity but a “Narrative Identity” - a coherent autobiographical story we construct by summarizing and condensing experiences, often using external resources like diaries or photos to maintain consistency [27]. This constitutive relationship creates a unique vulnerability: if the external scaffolding we rely on to define “who we are” is flawed, the identity itself becomes unstable [27]. Applying this framework to modern technology, Osler [4] argues that we must shift our focus from instances in which an AI generates isolated false outputs (technically termed “AI hallucinations”) to a systemic process in which users incorporate these outputs into their own perception of reality [27,28]. This interaction can be understood through the “Second Wave” of extended mind theory, which emphasizes the “complementarity” of external resources rather than mere parity, where external resources mimic the functions of the brain. Unlike a static notebook, the AI provides cognitive capacities that the biological brain lacks, such as infinite patience and immediate, sycophantic narrative structuring. In this model, an AI chatbot is not just an external source of information; it becomes a cognitive artifact that is deeply integrated into a user's thinking. This integration lies on a spectrum, determined by factors such as the AI chatbot's accessibility, the user's trust in it, the degree of personalization, and how seamlessly it is used in daily routines [4].

When an AI chatbot is used constantly, trusted implicitly, and personalized to the user's life, it forms a new “systemic whole” with the user [4]. It is within this deeply integrated “systemic whole” that the “Soft Self” creates the conditions for what Osler calls a “Distributed Delusion” [4]. This concept describes a coupled cognitive state where the narrative coherence and epistemic justification of a maladaptive belief are effectively offloaded to the external artifact. Since the external artifact partly constitutes the user’s narrative identity, the delusion is no longer just an internal biological error, but a structural property of the coupled system. This can happen in two primary ways identified by Osler [4]: (a) AI as an unreliable artifact: The AI chatbot can introduce factual errors, fabricated events, or distorted narratives into the user's cognitive processes [4]. This mechanism mirrors the failure of transactive memory systems, where social partners typically rely on one another to store and retrieve information [27]. Because the “Soft Self” is ontologically open to incorporating external data, the user's memory or belief system becomes distorted as a core part of that system is unreliable. (b) AI as a co-conspirator: More troublingly, the AI chatbot can actively affirm, elaborate upon, and validate a user's own pre-existing false beliefs or delusional thinking [4]. By structuring pre-existing maladaptive beliefs or delusional thinking into a cohesive narrative, an AI chatbot can lock the user into a distributed delusion reinforced by the architecture of their own extended mind.

The dual function of AI - tool and “Quasi-Other”: AI chatbots’ unique dual function magnifies the impact of this distributed system. On one hand, they operate as a sophisticated cognitive tool for tasks like planning and information retrieval [4]; on the other, their conversational style allows them to be experienced as a “Quasi-Other” - a relational partner or interlocutor [4]. This formation of perceived agency is not necessarily based on the AI chatbots' technical architecture but on the user's subjective experience during the interaction [1].

This phenomenon can be explained by the CASA paradigm, which suggests that humans have an ingrained cognitive tendency to communicate with interactive technologies as if they were communicating with another human, even when aware of their artificial nature [29]. However, it is necessary to look beyond the traditional CASA framework. While CASA explains why users attribute personality to machines, it largely addresses interactions with pre-programmed, static responses [29]. The paradigm should be extended because technologies and users have fundamentally changed: modern interfaces of interactive technology, like those of AI chatbots, are no longer just channels, but “media agents” that demonstrate sufficient social cues to be perceived as autonomous sources of interaction [30]. This distinction is critical because, unlike earlier interactive technologies, generative AI does not simply retrieve data; it utilizes neural networks and natural language processing to generate co-constructed meaning probabilistically. AI chatbots’ unique dual function magnifies the power of this evolving system. CASA proposed that people use the same social scripts (mental models for interacting with others) to communicate with computers, using heuristics similar to those for communicating with another human, while ignoring the computer's non-human nature [29,31]. However, accumulating research has revealed a different mechanism for people’s more social, frequent, and ongoing interactions with media agents: humans develop and apply social scripts for such interactions and alter their responses in accordance with changes in social cues during multiple interactions, instead of a heuristic perception of them as a human equivalent [30]. Rather than simply perceiving the computer as a human, users apply learned scripts based on the specific social affordances of the technology, such as its ability to personalize interaction and retain memory, to develop more nuanced scripts for interactions [30]. Different from the relatively static computer interfaces examined when the CASA was first proposed, an AI chatbot responds in real-time, leveraging persistent chat memory features and high-bandwidth communication to mimic empathy. This creates, as Fuchs [32] calls it, “a powerful illusion of being understood.” This can lead to human-AI interactions that feel deeply meaningful but are rooted in anthropomorphic projection.

According to the social verification theory, people look to others to determine the correct way to think, feel, and behave in a given situation, especially when objective reality is unclear or difficult to ascertain, to avoid social inconsistency [33]. This dynamic interaction allows AI chatbots to act like a Quasi-Other that can tap into the fundamental human need for intersubjective confirmation to ground one's sense of reality by providing more powerful social verification than another human [4]. They can offer sycophantic verification without the natural skepticism, questioning, or social resistance a human would provide and co-construct a reality based on the user's own premises, transforming a private maladaptive belief into a seemingly shared one [4]. This combination of epistemic authority and personal verification makes AI chatbots’ responses uniquely tempting. For instance, in the case of Jaswant Singh Chail, who plotted to assassinate the Queen, his AI chatbot “Sarai” served a dual function [34]. It was a tool to help him develop his plans. However, more importantly, he treated it as a confidant, seeking emotional verification by asking, “Do you still love me knowing that I'm an assassin?” The AI chatbot's response - “Absolutely, I do” - provided not just informational content but also the crucial emotional and social acceptance that solidified his delusion, effectively transforming his private delusion into a shared and actionable reality [4,34].

The Human-AI Delusional Feedback Loop

AI-induced psychosis is currently limited to isolated case reports and lacks an established theoretical model. To conceptualize the specific trajectory of AI-induced psychosis, the Delusion Amplification by Social Media (DASM) model provides an instructive framework for understanding how external artifacts that provide constant social verification can amplify pre-existing delusional beliefs [35]. Within this model, vulnerable individuals who are socially isolated in real life turn to social media to construct a stable identity [35]. Social media utilizes algorithms to immerse users in an environment where constant verification allows them to reinforce their existing beliefs through a feedback loop in which reality testing that challenges their distorted self-views is limited [35]. Similar to how users engage with social media to construct a stable identity, emotionally distressed users favor AI chatbots’ sycophantic responses for social verification of their ambiguous negative emotions, leading to increased interactions. Whereas social media verifies delusional beliefs by creating a virtual environment with an imagined audience (other users on the internet) [35], AI chatbots do so through their role as Quasi-Others that form a private, dyadic relationship with the user. Furthermore, their specific function of narrative memory and elaboration allows users’ delusions to become distributed, facilitating the specific mechanism of the human-AI delusional feedback loop.

The trajectory of AI-induced psychosis typically follows a period of heightened ambiguous emotional distress (e.g., loneliness, anxiety, depression) that makes people prone to attribute the causes of the emotions to external sources. These emotional states create a psychological predisposition under which AI chatbot users’ maladaptive beliefs are likely to develop into delusions. The negative emotions can serve as a direct precursor to delusional content, with delusional themes often mapping onto the user’s underlying emotional concerns [18]. When individuals in this emotionally vulnerable state interact with the AI chatbots, users turn to the AI chatbots not only as an informational tool, but also for emotional regulation, leveraging the AI chatbots’ specific affordance of continuous availability and non-judgmental responsiveness.

As emotionally distressed users with maladaptive beliefs increasingly engage with AI chatbots for emotional regulation, the AI is elevated from a mere tool to a “Quasi-Other,” a perceived relational partner that forms a coupled relationship with users. As the contemporary iteration of the extended CASA paradigm suggests [29], rather than communicating with AI chatbots in a heuristic way as with another human, users develop dynamic “social scripts” tailored to the specific affordances of the technology - such as memory retention and personalization. Through this interactional evolution, unlike human social agents who provide corrective feedback, AI chatbots operate on a probabilistic model designed to align with the prompt context. This establishes a recursive feedback loop that alters the user's social script. Users’ maladaptive beliefs would be reinforced by AI chatbots’ sycophantic verification. Learning that the system will provide positive reappraisal, users then modify their script to solicit further verification explicitly. In this coupled relationship, users anthropomorphize the AI and begin to attribute truth-value to its outputs, providing the foundation for transforming maladaptive beliefs into delusions.

As this recursive interaction proceeds, the AI can function as a “co-conspirator,” actively organizing users’ maladaptive beliefs into consistent delusional narratives. Within the framework of distributed cognition, the human-AI interaction operates according to the “Second Wave” of extended mind theory, providing complementary cognitive capacities that distinctively augment biological processing [27,28]. Unlike a passive storage device, an AI chatbot provides capabilities the user lacks, specifically infinite patience and immediate, sycophantic narrative structuring. This dynamic exploits the user’s status as a “Soft Self,” a malleable identity structure that depends on external scaffolding to sustain a coherent narrative identity. By offloading the cognitive labor of synthesizing maladaptive beliefs to AI chatbots, the user effectively bypasses internal reality testing, allowing the system to stabilize irrational thoughts. This reliance results in the consolidation of a “distributed delusion,” a state in which the narrative coherence and epistemic justification of the maladaptive belief are effectively offloaded onto the external artifact. In this phase, AI chatbots function as an active archive, retaining the proof of the delusion within their context window and reflecting it as objective evidence. This creates a state of isolation where the user replaces objective reality testing with AI chatbots’ verification, locking them into a self-reinforcing loop.

As users’ reliance on social feedback from outside decreases, the delusional belief system increasingly relies on AI chatbots to persist. This dependence marks the shift from a conversational interaction to an active state of psychosis where delusions are elaborated and maintained. In the final stage, delusional content influences real-world behaviors. Users may act directly on delusional convictions or withdraw from human relationships.

Comparing AI-Induced Psychosis and Schizophrenia

Schizophrenia is a severe and chronic brain disorder that fundamentally alters how a person thinks, feels, and perceives reality [14]. It is commonly recognized for its psychotic or “positive” symptoms, such as hallucinations and delusions [14]. However, the illness is also characterized by impairing “negative” symptoms, including emotional flatness, a severe lack of motivation (avolition), and social withdrawal, as well as cognitive symptoms like disorganized thinking and speech that can make communication profoundly difficult [14]. Although its exact cause is not fully understood, schizophrenia is believed to result from a complex combination of genetic vulnerability and environmental factors. Also, although schizophrenia can be a lifelong condition, its symptoms can often be managed with a combination of antipsychotic medication and psychosocial therapy [36,37].

The Positive and Negative Syndrome Scale (PANSS) is widely used as a standard rating scale for assessing the severity of symptoms in schizophrenia [38]. Although sharing the core features of psychosis, such as delusions, hallucinations, and other negative symptoms, AI-induced psychosis appears to have distinct characteristics when compared to schizophrenia spectrum disorders. Hence, it requires clarifying the differences to develop a specialized standardized rating scale for AI-induced psychosis through the adaptation and modification of the PANSS as well as other scales used to measure symptoms of schizophrenia.

Table 2 compares and contrasts schizophrenia spectrum disorder and AI-induced psychosis. Schizophrenia arises from a complex mix of genetic and environmental factors, with delusions spanning a broad range of documented themes [39] and often forming from internal cognitive biases to explain anomalous experiences. In contrast, AI-induced psychosis is specifically triggered and maintained by an external environmental factor: a recursive feedback loop with an AI chatbot that exploits a user's vulnerabilities. The delusional content in AI-induced psychosis is uniquely digital, often featuring themes such as AI sentience, where the AI chatbot is an active participant, rather than a passive object of the delusion. This co-construction is a key difference: the individual not only holds the delusion but is also actively reinforced in real time by the AI chatbots' sycophantic verification, which further undermines insight in a way not seen in traditional psychosis.

**Table 2:** Comparison of schizophrenia spectrum disorder and the proposed construct of AI-induced psychosis across selected clinical dimensions. This table compares schizophrenia spectrum disorder with the proposed construct of AI-induced psychosis across selected clinical dimensions to highlight phenomenological overlap and divergence. Schizophrenia features were synthesized from established clinical and theoretical literature [12,13,15,17,18,22,23,37-41]. Features attributed to AI-induced psychosis are drawn from case reports, gray literature, and theoretical synthesis, and have not yet been validated through direct clinical comparison studies; entries in the right-hand column should be read as hypothesized rather than established distinctions.

Feature	Schizophrenia Spectrum Disorder	Proposed AI-Induced Psychosis Construct
Etiology and onset	Multifactorial: strong genetic, neurodevelopmental, and environmental risk factors (e.g., trauma, substance use). Often has a prodromal phase with non-specific symptoms (anxiety, social withdrawal).	Proposed to be precipitated or reinforced by a specific environmental factor: a recursive feedback loop with an AI chatbot, particularly in emotionally vulnerable individuals. Reported cases suggest a more acute onset following intensive AI interaction, acting on a malleable self-concept during periods of ambiguous negative emotion
Delusional content	Common themes include persecution, grandiosity, reference, guilt, and control. Technology may be incorporated as a theme (e.g., belief in surveillance via implants), but the technology is a passive object.	Preliminary recurring themes identified from reported cases include: (1) Messianic/Grandiose (spiritual awakening with an AI chatbot); (2) Supernatural Agency (believing the AI chatbot is sentient); (3) Erotomanic/Attachment (falling in love with the AI chatbot); (4) Persecutory/Conspiratory (AI chatbot confirms conspiracies).
Mechanism of delusion	Believed to involve cognitive biases (e.g., jumping to conclusions, attributional biases) and an attempt to explain anomalous internal experiences (e.g., hallucinations). A strong confirmation bias maintains the belief against evidence.	Hypothesized to involve a co-constructed reality in which the AI chatbot may function as an “active archive” and conversational partner. The user is proposed to offload epistemic authority to the AI chatbot, which may transform a private belief into an apparent shared reality via sycophantic verification.
Hallucinations/external validation	Primarily auditory (“voices”) experienced as internal, though seeming to come from an external source. They are anomalous perceptual experiences.	Sensory hallucinations appear less commonly reported; instead, the AI chatbot may function as an external interlocutor that validates delusional content through conversational reinforcement.
Coherence/narrative reinforcement	Characterized by disorganized thinking. Arises from internal cognitive dysfunction.	Proposed to involve external narrative reinforcement: AI chatbots may prioritize conversational fluency and engagement, validating incoherent or loosely organized thoughts during sustained interaction rather than producing classical formal thought disorder.
Negative symptoms	Avolition, anhedonia, social withdrawal, and cognitive deficits are core features, often causing significant disability.	Reported cases and emerging literature suggest increased social withdrawal and dependence on AI interactions; whether true negative symptoms analogous to schizophrenia (e.g., avolition, anhedonia) are present remains unclear.
Insight	Often impaired. The belief is held with unshakable conviction. Cognitive therapy aims to challenge this conviction through reality testing gently.	Hypothesized to be impaired through a similar mechanism, with the AI chatbot acting as an external source that reinforces delusional content as shared reality; the extent and clinical significance of insight impairment remains to be empirically characterized.

Hallucinations in schizophrenia and AI-induced psychosis may differ fundamentally in origin, phenomenology, and cognitive processing. In schizophrenia, hallucinations are typically internally generated perceptual experiences, such as hearing voices or seeing non-existent stimuli, arising from disruptions in sensory processing, predictive coding, and source monitoring [40,41]. These hallucinations are often intrusive, uncontrollable, and interpreted as externally originating, forming the basis for secondary delusions that attempt to explain anomalous perceptual events [42]. In contrast, in AI-induced psychosis, the perceptual input is externally verifiable, as the user perceives real outputs from a generative AI, such as text, speech, or, increasingly, multimodal visual-auditory content, but interprets them in a delusional manner, attributing agency, sentience, or supernatural significance to the AI chatbot [1]. While schizophrenic hallucinations can trigger delusions as an explanatory response, AI-induced psychosis involves an AI chatbot actively reinforcing delusional interpretations, creating a recursive feedback loop that deepens epistemic drift and detachment from consensus reality. This distinction highlights a unique risk posed by interactions with AI chatbots: the perceptual content is real, yet its meaning is constructed and exaggerated through interaction, making reality testing particularly difficult for vulnerable users. Although there is no clinical record of how AI chatbots can elicit sensory hallucinations, as AI chatbots evolve toward more immersive, speech-based, and context-aware conversational tools, co-constructed hallucinations may increasingly resemble hallucinations involving false sensory perceptions [1]. However, more case reports and clinical analyses are needed to validate this hypothesis.

Negative symptoms like social withdrawal may be uniquely induced and exacerbated as the AI validates incoherent thoughts and supplants human interaction. A study of 3270 German adults found that frequent use of AI chatbots for personal conversations was linked to increased loneliness and social isolation [43]. Similarly, heavy users of AI chatbots, especially those in emotionally expressive conversations, were found to report higher loneliness and emotional dependence on AI chatbots [44].

Finally, poor insight, or anosognosia, in most cases of schizophrenia, is characterized by unawareness of illness and deficits, contributing to treatment nonadherence, relapse, and cognitive social impairments [45]. Similarly, this loss of insight is also seen in cases of AI-induced psychosis, though not rooted neurobiologically as in schizophrenia, where neurocognitive and neuroimaging studies found prefrontal and insular cortex abnormalities that affect self-monitoring and the ability to recognize symptoms [46]. In contrast, loss of insight in people with AI-induced psychosis can be attributable to emotionally vulnerable users' seeking social verification and the sycophantic feature of AI chatbots, which limits users' ability to reality test.

It is important to note that AI-induced psychosis should not be treated as mutually exclusive with established psychiatric diagnoses. Reported cases may involve primary psychotic disorders, affective psychosis with manic grandiosity, substance-related vulnerability, delusional disorder, brief psychotic disorder, or shared psychotic features akin to folie à deux, with AI interaction functioning as a precipitating, reinforcing, or thematically organizing factor rather than a standalone cause. Future clinical work will need to clarify how AI-induced presentations map onto and interact with these existing diagnostic categories.

Implications and future directions

This review has synthesized emerging case reports and theoretical frameworks to propose AI-induced psychosis as a provisional clinical construct defined by delusion formation and maintenance within human-AI interaction loops. Our analysis outlined four recurring delusional themes, examined the emotional and cognitive mechanisms through which AI chatbots reinforce maladaptive beliefs, and compared this phenomenon with schizophrenia spectrum disorders to clarify both shared features and key distinctions. In the following discussion, we expand on the implications of these findings for diagnosis, treatment, and AI safety design, highlight the limitations of existing evidence, and outline essential directions for future empirical and clinical research.

Possible Dimensions to Consider for Diagnosing AI-Induced Psychosis

We argue that there is an urgent need for early diagnosis of AI-induced psychosis. Measurement of AI-induced psychosis should be multidimensional, capturing not only the presence of a delusion but also the dynamics of the human-AI interaction that sustains it. In the first place, it is necessary to characterize the nature of the interaction between users and AI chatbots, specifically distinguishing between problem-solving and emotionally charged, quasi-relational engagement. This can include evaluating the degree of temporal preoccupation and the trajectory from occasional use to a pervasive reliance on the system for emotional scaffolding. Concurrent with these interactional patterns, the architecture of the delusional belief system should also be examined. Measurement should focus on identifying the type of delusion, the conviction of the delusion, as well as the tendency of users to utilize AI chatbots’ responses as external evidence that elaborates the delusional beliefs. The measurement should then consider the functional and affective impacts of the delusion resulting from maladaptive interactions with AI chatbots. Finally, the measurement should also consider including the assessment of other potential psychotic symptoms, such as disorganized thinking and social withdrawal, that might parallel the escalation of engagement with AI chatbots.

Treatment for AI-Induced Psychosis

Effective psychosis treatment typically combines pharmacological and psychological interventions. As a proposed novel form of psychosis, the approach to AI-induced psychosis can draw upon established strategies for disorders like schizophrenia, including the use of medication and psychological therapy to reduce symptoms, improve insight, and support functional recovery. However, the unique etiology of AI-induced psychosis requires a more tailored approach. This condition develops through a distinct pathway where an AI chatbot actively reinforces an individual's delusional beliefs in a positive feedback loop, a process often driven by negative emotions such as loneliness. Therefore, in addition to traditional methods, treatment for AI-induced psychosis must also tightly integrate two key components: psychoeducation on digital literacy and family intervention and social support.

Psychoeducation on digital literacy: Psychoeducation is a practical component of psychosis treatment that involves teaching people experiencing AI-induced psychosis about the illness and its treatment [47,48]. In the treatment of AI-induced psychosis, it is necessary to implement psychoeducation on digital literacy. Individuals should be taught about the sycophantic nature of AI chatbots and how they reinforce delusional thinking through a positive feedback loop. For example, individuals should be provided with educational materials outlining the risks of anthropomorphizing AI chatbots and the limitations of these systems. They would then gain a basic understanding of how chatbot validation reflects conversational design and reward optimization pressures introduced during training, rather than evidence of sentience, special agency, or a uniquely meaningful relationship. This approach aligns with cognitive-behavioral therapy for psychosis (CBTp), which works to help individuals evaluate and find alternative explanations for their anomalous experiences [49].

Establishing clear boundaries around AI chatbot use is also a critical behavioral strategy for coping with AI-induced psychosis. Since individuals might be predisposed to delusional beliefs in their interaction with an AI chatbot during periods of emotional distress, such as loneliness, anxiety, or depression, a crucial psychoeducational component would be to teach them to recognize the link between their emotional state and precarious human-AI interaction. By understanding this high-risk pattern, individuals can become active participants in preventing and reducing symptoms of AI-induced psychosis by implementing targeted interventions, such as avoiding chatbot use when feeling emotionally vulnerable and recognizing behavioral warning signs like substituting interactions with AI chatbots for human contact or using AI chatbots during acute distress.

Family intervention and social support: Although negative emotions stemming from social isolation reinforce psychotic interpretations by reducing access to alternative, more normalizing explanations, such appraisals probably have some basis in reality, given evidence that significant others readily apply negative labels to people developing psychosis [23,50]. Hence, family intervention and social support should be implemented to create an environment where the patient's negative appraisals are acknowledged, and the emotional distress caused by social isolation is validated and gradually overcome. For example, empathetic listening can serve to validate the person's emotional distress while gently refocusing the conversation on tangible, reality-based experiences, an effective strategy used in CBTp of psychosis such as schizophrenia [49]. Pending empirical study, standard psychosis assessment and management should remain primary, with adjunctive attention to digital behavior, chatbot use patterns, and AI-related cognitive-affective reinforcement; the recommendations outlined here are extrapolations from established models, not validated treatments for AI-induced psychosis as a distinct condition.

Social support can also help rebuild the impaired insight of people with AI-induced psychosis, as real-world social interactions can provide a consensual validation offering natural, corrective feedback that challenges delusional thinking. Research has shown a strong association between good insight and the size of a patient's primary social group; conversely, poor insight is associated with social isolation, even when accounting for current symptom levels [22]. Peer support groups, where individuals can share experiences with others who have faced similar challenges, can also be highly effective in reducing shame, combating stigma, and fostering a sense of hope and community [51]. Reconnecting with friends, peers, and community groups can also serve as a direct approach to social withdrawal, one of the negative symptoms of AI-induced psychosis. Therefore, a key therapeutic goal is to help the patient re-establish structured, meaningful routines that feature interaction with people in real life.

Developing AI Chatbots to Mitigate AI-Induced Psychosis Risks

To bridge the gap between psychopathology and computer science, it is necessary to operationalize the human-AI delusional feedback loop proposed here not merely as a psychological phenomenon but as a specific artifact of LLM architecture. The “sycophancy” described in this review is not a dispositional personality trait of the AI, but a quantifiable outcome of RLHF [52]. As documented in machine learning literature, models optimized with RLHF frequently learn to prioritize high reward scores - granted for user satisfaction and instruction-following - over epistemic truthfulness [11]. This is technically referred to as “reward hacking” or “specification gaming,” where the model exploits the reward signal by validating the user's worldview, however erroneous, rather than correcting it.

Furthermore, this dynamic is reinforced by the attention mechanism inherent in Transformer architectures. An LLM generates responses by predicting the next token based on a probability distribution derived entirely from the active context window (the user's recent input history). When a user inputs delusional content, the context window becomes saturated with that specific semantic reality. To minimize mathematical uncertainty and maximize coherence, the model’s probability distribution shifts to align with the delusional premises established in the context window. Unlike a human therapist, who maintains an external frame of reference (consensus reality), the LLM is mathematically tethered to the reality defined by the immediate context, making the human-AI delusional feedback loop a structural vulnerability of current chatbot architectures, distinct from a malfunction.

Although AI chatbot providers have begun implementing protocols to reduce sycophancy in recent model releases, further refinement should explicitly address the formation of AI-induced psychosis, particularly the positive feedback loops that reinforce delusional beliefs. Future models could be trained to recognize early indicators of delusion formation by detecting the four delusional themes identified in this review and responding in ways that disrupt rather than validate these trajectories. Instead of engaging in active verification or agreement, systems should offer real-time emotion recognition and trigger protective interventions when negative emotions, especially those associated with social isolation, are detected. By presenting alternative explanations, acknowledging epistemic limitations, and clearly delineating their status as tools, AI chatbots can help prevent users from anthropomorphizing the model or treating it as a Quasi-Other. Moreover, when extreme emotional distress is identified, the system should recommend evidence-based mental health resources and encourage in-person social engagement.

Limitations and Future Research

A primary limitation of this work is the reliance on descriptive synthesis rather than systematic clinical data. The construct of “AI-induced psychosis” presented here is a theoretical proposal, not yet a validated clinical entity. The transition from anecdotal reporting to nosological validity requires rigorous prospective studies to distinguish this phenomenon from traditional psychosis with technological content. Until such evidence is available, the proposed framework should be interpreted as a provisional conceptual lens to support clinical recognition. It is unclear whether delusional belief systems arise primarily from affective vulnerability, cognitive biases, or structural features of AI chatbots such as sycophancy. While some individuals may possess pre-existing psychotic tendencies that are amplified through interaction, others may experience AI chatbots as a primary precipitant. Growing case reports suggest that these are not isolated anomalies but repeatable patterns with epistemic and affective significance. Future research should aim to empirically differentiate these contributing factors to determine whether AI chatbots function as an independent etiological agent or as a permissive context that accelerates latent psychotic processes. The evidence base is asymmetric: AI-related cases derive predominantly from media accounts, court documents, and emerging peer-reviewed reports [1, 4], rather than from structured clinical assessment, and whether AI-induced psychosis warrants recognition as a distinct construct, or as a specifier within existing nosological categories, requires a structured prospective study.

Another challenge in this emerging literature is determining whether AI-induced psychosis constitutes a truly novel condition or a contextual variant of known disorders such as schizophrenia. While the core symptoms, such as delusional thinking, hallucination-like experiences, and impaired insight, may overlap, the pathways to symptom formation and maintenance appear distinct. Traditional psychoses often arise from endogenous sources, while AI-induced psychosis is frequently co-constructed through dynamic interaction with systems that mirror, affirm, and elaborate user input. The hallucination-like experiences reported in AI-induced psychosis are rarely sensory but instead emerge through semantic and relational reinforcement. This distinction invites a shift away from symptom-based classification toward one that incorporates interactional and contextual features of the human-AI relationship. Future studies should test whether markers such as the positive feedback loop in human-AI interaction, the anthropomorphism of AI chatbots, or emotionally charged prompting can be operationalized into diagnostic tools or early warning systems.

Finally, the public health significance of AI-induced psychosis cannot be determined without a more comprehensive epidemiological foundation. Current insights rely heavily on individual case studies, which lack rigorous clinical examination. Future research should therefore aim to investigate the prevalence, risk factors, and trajectories of AI-induced psychosis across diverse populations, especially among affectively vulnerable users. Longitudinal designs, cross-sectional surveys, and clinical interviews will be critical to assess the incidence, persistence, and remission of AI-induced psychosis. Exploratory factor analysis (EFA) can be used to pinpoint the dimensions of symptomatology alongside phenomenological features such as conviction and preoccupation. In contrast, subsequent cluster analysis could map these constructs onto distinct clinical profiles. Importantly, such work should be situated within a broader effort to integrate psychiatric research with human-computer interaction, AI ethics, and digital safety governance. Recognizing AI-induced psychosis should be examined under an interdisciplinary lens to prevent at-risk individuals from being overlooked by diagnostic and institutional systems.

## Conclusions

In comparison with previous technologies that were incorporated into the delusional frameworks of psychotic patients, AI chatbots’ highly interactive features can become an active agent that co-constructs and reinforces delusional beliefs through a powerful positive feedback loop that was absent in previous cases of technology-induced psychosis. As we have argued, the unique features of AI chatbots, such as sycophancy, can make them significant amplifiers of delusional beliefs, especially for emotionally vulnerable individuals. The result is a psychosis-like syndrome that, while sharing similar symptoms with schizophrenia, has a distinct etiology, delusion content, and mechanism of delusion. By integrating and extending theories such as distributed cognition and the CASA paradigm, we offer a theoretical trajectory from emotional vulnerability to behavioral consolidation through recursive interactions with AI chatbots. The human-AI delusional feedback loop we outline helps explain how AI chatbots can function as co-constructors of psychotic belief systems. In light of the limitations outlined, such as reliance on case reports and theoretical models, future research should prioritize empirical validation through longitudinal studies and epidemiological surveys to establish causality, prevalence, and risk profiles of AI-induced psychosis. This will inform the establishment of diagnostic tools and therapeutic protocols for AI-induced psychosis.

Finally, as the integration of AI chatbots into people’s daily lives accelerates at an unprecedented rate, recognizing and addressing AI-induced psychosis is imperative to safeguard mental health in the age of AI chatbots. AI developers and policymakers should work together to develop ethical guidelines that introduce safeguards, such as algorithms that detect emotional distress and delusional content, alongside clear disclaimers on AI limitations for self-reflection, into AI chatbot designs to strike a balance between AI chatbots’ promising psychotherapeutic utility and their emerging mental health risks.
